# Determining Risk Factors That Affect Progression in Patients with Nonproliferative Diabetic Retinopathy

**DOI:** 10.1155/2021/6064525

**Published:** 2021-11-30

**Authors:** Dalbert J. Chen, Jacky C. Kuo, Alex J. Wright, Alice Z. Chuang, Wenyaw Chan, Robert M. Feldman, Eric L. Crowell

**Affiliations:** ^1^Ruiz Department of Ophthalmology and Visual Science, McGovern Medical School at The University of Texas Health Science Center at Houston (UTHealth), 6431 Fannin St., MSB 7.204, Houston, TX 77030, USA; ^2^Department of Biostatistics and Data Science, School of Public Health, The University of Texas Health Science Center at Houston (UTHealth), 1200 Pressler St., RAS E827, Houston, TX 77030, USA; ^3^Robert Cizik Eye Clinic, 6400 Fannin St., Suite 1800, Houston, TX 77030, USA; ^4^Lyndon B. Johnson Hospital, Harris Health, 5656 Kelley St., Houston, TX 77026, USA

## Abstract

**Purpose:**

To determine risk factors that affect nonproliferative diabetic retinopathy (NPDR) progression and establish a predictive model to estimate the probability of and time to progression in NPDR. *Patients and Methods*. Charts of diabetic patients who received an initial eye exam between 2010 and 2017 at our county hospital were included. Patients with proliferative diabetic retinopathy (PDR), fewer than 2 years of follow-up, or fewer than 3 clinic visits were excluded. Demographics and baseline systemic and ocular characteristics were recorded. Follow-up mean annual HbA1c and blood pressure, best-corrected visual acuity, and the number of antivascular endothelial growth factor treatments were recorded. Stage and date of progression were recorded. A 5-state nonhomogeneous continuous-time Markov chain with a backward elimination model was used to identify risk factors and estimate their effects on progression.

**Results:**

Two hundred thirty patients were included. Initially, 65 eyes (28.3%) had no retinopathy; 73 (31.7%) mild NPDR; 60 (26.1%) moderate NPDR; and 32 (13.9%) severe NPDR. Patients were followed for a mean of 5.8 years (±2.0 years; range 2.1–9.4 years). 164 (71.3%) eyes progressed during the follow-up. Time-independent risk factors affecting progression rate were age (hazard ratio (HR) = 0.99, *P*=0.047), duration of diabetes (HR = 1.02, *P*=0.018), and Hispanic ethnicity (HR = 1.31, *P*=0.068). Mean sojourn times at mean age, duration of diabetes, and annual HbA1c for a non-Hispanic patient were estimated to be 3.03 (±0.97), 4.63 (±1.21), 6.18 (±1.45), and 4.85 (±1.25) years for no retinopathy, mild NPDR, moderate NPDR, and severe NPDR, respectively. Each 1% increase in HbA1c annually diminished sojourn times by 15%, 10%, 7%, and 10% for no retinopathy, mild NPDR, moderate NPDR, and severe NPDR, respectively.

**Conclusion:**

HbA1c level is a significant modifiable risk factor in controlling the progression of DR. The proposed model could be used to predict the time and rate of progression based on an individual's risk factors. A prospective multicenter study should be conducted to further validate our model.

## 1. Introduction

Diabetic retinopathy (DR) is the leading cause of blindness in the United States [1]. Vision loss in DR is often due to diabetic macular edema [2], abnormal blood vessel growth, or retinal scarring [3, 4]. DR is classified into 4 clinical stages: mild nonproliferative diabetic retinopathy (NPDR), moderate NPDR, severe NPDR, and proliferative diabetic retinopathy (PDR) [5]. The 3 NPDR stages have varying degrees of vascular permeability, capillary occlusion, and retinal vasculature abnormalities, in the form of microaneurysms, hemorrhages, hard exudates, cotton wool spots, and venous beading [6].

NPDR progression can lead to PDR. PDR is characterized by neovascularization due to retinal hypoxia, which stimulates vascular endothelial growth factor (VEGF) production [7, 8]. Patients who have PDR can experience severe vision impairment when abnormal vessels bleed into the vitreous or when tractional retinal detachment occurs from fibrous scarring [3, 4]. With the prevalence of diabetes projected to increase to more than 54 million Americans by 2030 [9], there is a growing potential for vision loss in this population. Minimizing vision loss by preventing the progression of DR could save vision in diabetic patients.

To slow the progression of NPDR to PDR, it is important to determine the risk factors associated with the progression of DR. Poor glycemic control as measured by increased hemoglobin A1c (HbA1c) level has been shown to be a significant risk factor associated with the progression of NPDR to PDR [10–13]. Other risk factors, such as patient age, sex, race/ethnicity, hypertension status, dyslipidemia, blood pressure (BP), and use of anti-VEGF therapy, have been assessed, but not all studies agree on which risk factors significantly affect the incidence or progression of DR [10–16]. Of the mentioned risk factors, only HbA1c, dyslipidemia, and BP are modifiable. However, the effects of HbA1c and BP on DR progression time and progression probability over time for each stage have not been studied.

The purpose of this study is to determine the risk factors, both time-independent and time-dependent, which affect the progression of DR and establish a predictive model that uses these risk factors to estimate an individual patient's progression probability and progression time to the next DR stages.

## 2. Patients and Methods

This retrospective chart review was conducted at Lyndon B. Johnson General Hospital (LBJ) of the Harris Health System and Robert Cizik Eye Clinic of the Ruiz Department of Ophthalmology and Visual Science at the McGovern Medical School at The University of Texas Health Science Center at Houston (UTHealth). Institutional Review Board approval was obtained from The University of Texas Health Science Center Committee for the Protection of Human Subjects (CPHS) and the Harris Health System. All research adhered to the tenets of the Declaration of Helsinki and was HIPAA compliant. The Institutional Review Board (CPHS) determined that informed consent was waived for this study. All categorizations in this study were reported using the data found in the patients' charts.

### 2.1. Study Population

All diabetic patients who received eye exams at the Ophthalmology Clinic at LBJ, a safety net hospital system, from January 1, 2010, to June 1, 2017, were identified by diabetes-related ICD-9 codes (250.00, 250.01, 250.50, and 362.01–362.07) and ICD-10 codes (E10.9, E10.3xx, and E11.3xx) and reviewed. Patients who were initially diagnosed with PDR, had retinal vein occlusion, had fewer than 2 years of follow-up, or had fewer than 3 clinic visits were excluded. No other concomitant diseases were excluded except for retinal vein occlusions. If both eyes were eligible, the right eye was included.

### 2.2. Treatment Management

Diabetes mellitus (DM) evaluations and medications were managed by the Primary Care service at Harris Health. HbA1c tests were ordered, and oral and/or injectable DM medications were prescribed accordingly by the patients' Primary Care physicians. The Ophthalmology service did not administer any ocular treatments at the NPDR stages besides anti-VEGF injections for diabetic macular edema (DME). When patients progressed to PDR, standard treatments, such as anti-VEGF injections, panretinal photocoagulation, and pars plana vitrectomy, were administered by the Ophthalmology service to prevent blindness.

### 2.3. Data Collection

Demographics (age at initial eye exam, sex, and race/ethnicity), baseline systemic characteristics (duration of diabetes, HbA1c level, insulin dependency, status of hypertension, systolic and diastolic BP, cardiovascular diseases, kidney diseases, amputations, and smoking status), and ocular characteristics (DR stage, presence of DME, best-corrected visual acuity (BCVA), and anti-VEGF treatment) were recorded. Mean annual HbA1C and BP, along with BCVA and number of anti-VEGF treatments, were calculated and recorded for each follow-up visit. If progression occurred, the stage and date of progression were recorded.

### 2.4. Measurements

Stages of DR were graded by ophthalmology residents of all levels as no retinopathy; mild, moderate, or severe NPDR; and PDR based on the Early Treatment Diabetic Retinopathy Study (ETDRS) classification criteria [5] with slit-lamp biomicroscopy and indirect ophthalmoscopy. All resident gradings were verified by a board-eligible/certified attending ophthalmologist. Snellen BCVA was converted to the logMAR scale, −log_10_ (BCVA), with the following adjustments: count finger (CF) was coded as 20/1500; hand motion (HM) as 20/4000; light perception (LP) as 20/8000; and no light perception (NLP) as 20/20000.

### 2.5. Data Analysis

Data were summarized using frequency (%) for discrete variables (i.e., sex, race/ethnicity, and presence of DME) and mean and standard deviation for continuous variables (i.e., age and HbA1c level). Demographics were compared among DR stages using the Chi-squared test or one-way analysis of variance (ANOVA) with Duncan multiple comparison. The rate of progression from each stage and time duration in each stage before progression were estimated using the continuous-time Markov chain (CTMC) model with transition (progression) matrix shown in Table 1, under the assumption that once the patient has progressed, they will be considered having progressed regardless of the possibility of regression. The model took several risk factors into consideration: age at an initial eye exam, sex, race/ethnicity, duration of DM, and baseline hypertension as the time-independent risk factors, and annual mean HbA1c, BP, and the number of anti-VEGF treatments as the time-dependent risk factors.

### 2.6. Continuous-Time Markov Chain Model

DR was categorized into 5 stages: (1) no retinopathy, (2) mild NPDR, (3) moderate NPDR, (4) severe NPDR, and (5) PDR [5]. Progression of DR was assumed to be irreversible (no regression) and that progression through more than one stage in a given time period could occur; for example, DR could directly progress from mild to severe without going through the moderate stage.

The risk factors of interest included time-independent factors and time-dependent factors. The effects of the risk factors were independent of the disease stages, such that the effects were the same for each DR stage. In addition, the time-dependent risk factors were constant between observation times (e.g., between year 1 and year 2, the HbA1c value was the same as the value obtained at year 1 visit).

### 2.7. Imputation of Missing Values

Missing annual HbA1c values during follow-up visits were imputed with the last known value, while missing baseline HbA1c values were imputed with the next known value. Missing values for the duration of DM were imputed by regressing the duration of DM on baseline HbA1c, baseline BP, and dyslipidemia status, chronic kidney disease, myocardial infarction, and cerebrovascular accident.

### 2.8. Nonhomogeneous Continuous-Time Markov Chain Model

A 5-state nonhomogeneous CTMC model was used to analyze the progression of DR. The CTMC model was used to estimate the progression rate matrix (Table 1) and the effects of risk factors (Supplementary Material S1) [17–19]. A backward elimination procedure using Akaike Information Criterion was performed to select the best set of risk factors to explain the dynamics of the DR progression model. It should be noted that not all risk factors remaining in the final model had a *P* value < 0.05. The “msm” R package (version 1.6.8) was utilized to obtain the maximum likelihood estimation of progression rates and the effects of risk factors [17].

### 2.9. Estimation of Sojourn Time

The sojourn time is the expected time to progress to the next stage. Since time-dependent risk factors are assumed to remain constant between observational times, the mean sojourn time at each stage was derived using a piecewise integration approach. This approach was convergent when the follow-up period and/or the number of follow-up visits was large (Supplementary Material S2). In this study, patients were followed for 2 to 10 years. To ensure that the piecewise approximation approach was converged adequately, we set a follow-up time for 20 years, and the values of time-dependent risk factors after the last visit were set to the last observed values.

### 2.10. Estimation of Transition Probability Matrix

Similarly, by the piecewise constant assumption, the progression probability matrix in our nonhomogeneous CTMC model, Pt1,tn, can be expressed as the product of several homogeneous segments (Supplementary Material S3).

All calculations were performed in R version 3.6.1. A *P* value < 0.05 was considered statistically significant.

## 3. Results

Two hundred and thirty (230) patients were included. There were 65 eyes (28.3%) with no retinopathy; 73 (31.7%) mild NPDR; 60 (26.1%) moderate NPDR; and 32 (13.9%) severe NPDR. Of these patients, 143 (62.2%) were female, and the majority of patients were Hispanic (158 (68.7%)), followed by Black (50 (21.7%)) and White (20 (8.7%)), race/ethnicity. The mean age at the time of the initial eye exam was 56.0 (±10.0) years. The mean age was 58.8 (±10.8, range 20–84) years for patients with no retinopathy; 55.2 (±10.5, range 21–76) years for mild NPDR; 54.8 (±8.3, range 37–77) years for moderate NPDR; and 54.5 (±9.6, range 26–72) years for severe NPDR. Although there was no difference in the mean age among the DR stages (*P*=0.067 using one-way ANOVA), the patients with no retinopathy were significantly older than the patients with severe retinopathy by Duncan's multiple comparison analysis.

### 3.1. Baseline Characteristics

The majority of patients had type 2 DM with a mean duration of diabetes diagnosis of 13.1 (±7.5, range 0.8–38.7) years. Mean baseline HbA1c level was 9.7% (±2.4%, range 5.4%–16.8%), and mean baseline BP was 135.6 (±18.2, range 91–205)/76.3 (±11.5, range 51–110) mmHg. Sixty-two (62, 27.0%) patients were insulin-dependent. The majority of patients had hypertension (184 (80.4%)) or hyperlipidemia (172 (74.8%)). Forty-three (43, 18.7%) patients had 1 or more severe systemic conditions secondary to DM, including chronic kidney disease (19 (8.6%)), amputation (12 (5.2%)), cardiovascular accident (8 (3.5%)), myocardial infarction (5 (2.2%)), and/or severe blockage requiring coronary artery bypass graft (7 (3.0%)). Of 19 chronic kidney disease patients, 15 were of stage 3. Demographics and baseline systemic characteristics are summarized in Table 2.

Overall mean BCVA was 0.45 logMAR (±0.54, range 0.0 to 2.6). At baseline, 39 eyes (17.0%) had DME. Of these eyes with DME, only 3 eyes had visually significant DME and were treated with anti-VEGF injections; 1 eye was treated with both triamcinolone and anti-VEGF injections. Seven eyes (7, 3.0%) had glaucoma. Of the 215 phakic eyes, 144 (66.5%) had cataracts. Two eyes (2, 0.9%) had concurrent hypertensive retinopathy. No age-related macular degeneration, retinal detachments, or retinal arterial occlusions were observed. Baseline ocular characteristics are summarized in Table 3.

### 3.2. Follow-Up Period

Patients were followed for an average of 5.8 years (±2.0 years; range 2.1 to 9.4 years). The overall average visits were 2.4 times (±1.7) with 3.0 times (±3.6) in no NPDR, 1.9 times (±1.1) in mild NPDR, 2.1 times (±1.7) in moderate NPDR, 2.0 times (±1.2) in severe NPDR, and 3.6 times (±1.7) in PDR. A total of 164 (71.3%) eyes progressed during the follow-up period, and 74 (of 230, 32.2%) eyes progressed to PDR. The incidence of DR progression was highest during years 2 and 3 (12 months to 36 months), with approximately 1 in 4 patients progressing each year (Table 4).

Table 4 summarizes the risk factors during the follow-up period. The average baseline HbA1c was 9.71% (±2.35%) and decreased over time (HbA1c = 8.16% (±0.93%) at year 10). The average systolic BP gradually increased, while the average diastolic BP decreased over time. The percentage of patients with DME increased over time.

### 3.3. Diabetic Retinopathy Progression

The time-independent risk factors investigated were age at an initial eye exam, duration of DM, sex, race/ethnicity, and hypertension status. Time-dependent risk factors investigated were annual HbA1c, systolic BP, diastolic BP, and the number of anti-VEGF treatments. After a backward elimination procedure using Akaike Information Criterion, the final CTMC model included time-independent risk factors of race/ethnicity, age, and duration of DM at the initial eye exam visit and 1 time-dependent risk factor, HbA1c. Sex, hypertension status, annual BP, and the annual number of anti-VEGF treatments did not significantly affect the progression of DR.

### 3.4. Time-Independent Risk Factors

Table 5 presents the effects of the time-independent risk factors included in the final model. With each additional year in age at the initial visit, the progression (transition) rate of DR was 0.99 times slower; in other words, there was a 1% (95% CI = (0.1%, 2.7%)) decrease in the DR progression rate per additional year of age at the baseline visit. The progression rates were 2% (95% CI = (0.0%, 3.9%)) higher if the duration of DM at the initial visit increased by 1 year. Although the patient's race/ethnicity was not statistically significant (*P*=0.068), ethnicity was an important factor, which affected our overall model. Progression rates of Hispanic patients were 31% (95% CI = (−3.1%, 76.7%)) higher than those of non-Hispanic patients.

### 3.5. Time-Dependent Risk Factors

HbA1c was the only time-dependent risk factor that affected the progression of DR. When HbA1c increased by 1% per year, the sojourn time was reduced by 15% (0.46 years) for patients with no retinopathy; 10% (0.47 years) for mild NPDR; 7% (0.46 years) for moderate NPDR; and 10% (0.47 years) for severe NPDR (Table 6). In other words, with each 1% increase in HbA1c level per year, a diabetic patient is predicted to progress 0.46 years faster from no retinopathy to a future stage; 0.47 years faster from mild NPDR to a future stage; 0.46 years from moderate NPDR to a future stage; and 0.47 years from severe NPDR to a future stage.

### 3.6. Sojourn Time

**Figure 1:**
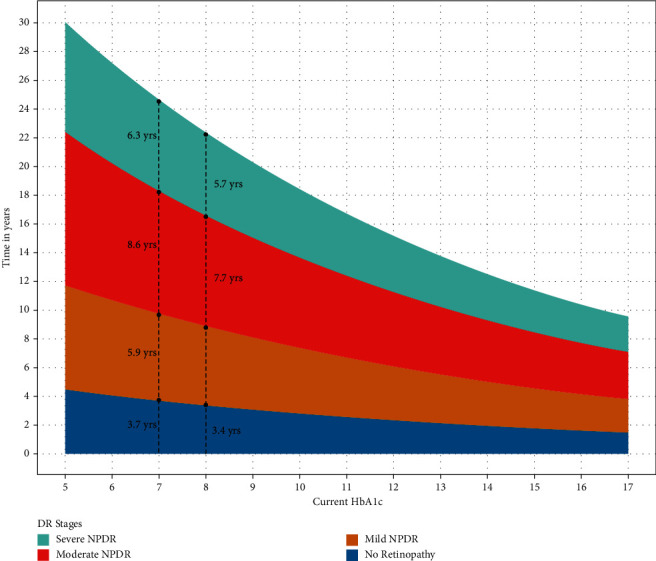
Illustration of the effect of HbA1c level on sojourn time, the expected time for a patient to progress to the next DR stage. For example, in our model, a patient who has mild NPDR with an HbA1c level of 7% is expected to progress to moderate NPDR in 5.9 years, while another patient who has mild NPDR with an HbA1c level of 8% is expected to progress to moderate NPDR in 5.4 years. DR = diabetic retinopathy; NPDR = nonproliferative diabetic retinopathy.

Sojourn time is defined as the expected time to progress to the next stage. The effect of HbA1c on the sojourn time for an individual study patient at a given time point is illustrated in Figure 1. For example, a patient who has mild NPDR with an HbA1c level of 7% is expected to progress to moderate NPDR in 5.9 years, while a patient with mild NPDR and an HbA1c of 8% is expected to progress to moderate NPDR in 5.4 years. It should be noted that Figure 1 illustrates the sojourn time for an HbA1c at a particular time point, which could be used for comparing 2 patients with different HbA1c levels at the given time. Table 6 provides the sojourn time reduction for a 1% increment of HbA1c annually for the same individual.

The estimated sojourn time for each study patient at each stage was calculated based on the patient's age, race/ethnicity, duration of DM, and annual HbA1c. The mean sojourn time was then calculated by averaging these individual sojourn times. The results were 3.03 (±0.97), 4.63 (±1.21), 6.18 (±1.45), and 4.85 (±1.25) years for no retinopathy, mild NPDR, moderate NPDR, and severe NPDR, respectively (Table 6).

### 3.7. Progression Rates

The estimated transition (progression) rate matrix was dependent on the values of risk factors.  Table 7 shows the estimated mean progression rate matrix over time, which takes the dynamic changes of HbA1c into consideration. The progression rates of a non-Hispanic individual with the age and duration of DM equal to the averages of the study cohort are presented. The progression rate matrix for Hispanic ethnicity can be obtained by multiplying the transition rates for non-Hispanics by 1.31 (see Table 5). Over time, the average rates of progression per year were 0.209, 0.142, 0.097, and 0.172 for no retinopathy to mild NPDR stage, mild NPDR to moderate NPDR stage, moderate NPDR to severe NPDR stage, and severe NPDR to PDR stage, respectively. The progression was slowest when a patient had moderate NPDR. This is shown in Table 6, where the highest sojourn time was for moderate NPDR at 6.18 (±1.45) years.

### 3.8. Probability of Progression to PDR

The probability of progression from each earlier stage to PDR within 1, 4, and 7 years is presented in Table 8. If the patient is at no retinopathy stage, the estimated probability of progression to PDR is 0.7%, 8.0%, and 20.6% within a 1-year, 4-year, and 7-year period, while a patient at severe NPDR stage has a 19.3% chance of progressing to PDR within 1 year, 56.4% within 4 years, or 76.4% within 7 years.

## 4. Discussion

This study used a nonhomogeneous CTMC model to estimate DR progression probability and the duration in each stage for a patient based on their risk factors. Our study creates a model based on age at the initial visit, annual HbA1c, and ethnicity to predict the rate of progression time through each ETDRS stage. This may influence clinical care by better predicting the required intervals for evaluating for change and, thus, the required frequency of office visits.

While current research and clinical practice indicate that HbA1c level is a risk factor for progression [10–13], our study is the first to investigate the effect of HbA1c on the progression rate and progression time for each ETDRS stage (PubMed search August 6, 2021, with a combination of terms nonproliferative diabetic retinopathy, progression rate, progression time, diabetes, and risk factors) [11]. Our model also identified the time-independent risk factors that affect the progression of DR. Included risk factors were age at the initial ophthalmology visit, duration of DM before the initial ophthalmology visit, and ethnicity (Hispanic versus non-Hispanic). When a patient's age was increased by 1 year at the time of the initial ophthalmology visit, the risk of progression was reduced by 1%. Additionally, the mean age of patients with no retinopathy was older than that of patients with severe retinopathy. Older age at initial presentation could be a protective factor against DR progression. One explanation for this reduction is that patients diagnosed with a late onset of DR have less duration of DR, and the longer a patient has diabetes, the higher the risk of developing advanced glycation end products, which could play a role in the development of DR [4].

Our patient demographic was 69% Hispanic with a mean duration of DM for 13.1 (±7.5) years before visiting an ophthalmologist. Although ethnicity and duration of DM were not significant risk factors individually, they did significantly affect the overall predictive model. Hispanic patients fared worse and were 31% more likely to progress compared to non-Hispanic patients. This may be an important factor that physicians should consider when deciding how closely to monitor a patient.

### 4.1. Risk Factors Affecting Progression

The Wisconsin Epidemiologic Study of DR (WESDR) analyzed 955 type 1 DM patients and followed them for 4 to 25 years. They concluded that progression of DR was more likely associated with less severe baseline DR, male sex, higher baseline HbA1c, an increase in the HbA1c level, and an increase in diastolic BP level from baseline to 4-year follow-up. Increased risk of progression to PDR was associated with more severe baseline DR, higher baseline HbA1c, greater baseline body mass index, and an increase in HbA1c between the baseline and 4-year follow-up examination [11]. The only risk factor for progression of DR shared between their study and ours was increased HbA1c; the other risk factors were not significant in our study. There are several reasons for the differences in findings. First, all the patients in the WESDR study had type 1 DM and were below 30 years of age, while only 2.2% of our study patients had type 1 DM and mean age 58.8 (±10.8). Second, WESDR used a 15-level DR staging classification, while we used the ETDRS 5-stage DR classification, which is the international clinical disease severity scale for DR [11, 14]. Third, WESDR had a larger sample size and longer follow-up time compared with our study. Lastly, WESDR used different models to investigate the risk factors associated with each patient's first progression of DR (any stage) and progression to PDR. Thus, their study showed inconsistent results between the 2 models. For example, they found that less severe DR at baseline was associated with progression of DR, while more severe DR at baseline was associated with progression to PDR. Our study used CTMC modeling, simultaneously identifying time-independent and time-dependent risk factors. In addition, the CTMC model has the added benefit of estimating the progression probability for each patient, given their risk factors.

Harris Nwayanwu et al. followed a cohort of 4,617 NPDR patients who were predominantly White (75%) for a median of 1.7 years and found that 307 (6.7%) progressed from NPDR to PDR during their study period. After adjusting for other risk factors (age, sex, race/ethnicity, etc.), their longitudinal Cox regression analysis with HbA1c as a time-dependent covariate showed that each 1% increase in HbA1c level was associated with a 14% increase in the hazard of progressing from NPDR to PDR [10], compared to our study which showed a 7% to 15% increase when HbA1c increases 1% annually depending on the stage. Similar to our findings, they also reported that the patients who progressed (mean age 57.3 years) were significantly younger than patients who did not progress (mean 59.5 years) at the initial diagnosis of NPDR. As with our data, there was no significant difference in sex in progression to PDR [10].

Several studies have noted a regression in DR stages when receiving anti-VEGF treatment for DME [15–19]. In our study, anti-VEGF treatment did not significantly affect progression rates of DR. The most notable difference is that our study looked at progression rates of patients with and without DME, whereas the above-mentioned studies evaluated only patients with DME. Compared to the Protocol W and Panorama studies [18, 19], our patients were from a safety net facility, were followed less rigidly, and received fewer injections. Thus, it appears important to follow the regimen reported in those studies to appreciate the regression effect. Additionally, our study also included non-DME patients who would not have received injections under the care protocols in place at the time of care, therefore reducing our sensitivity to improvement. This, in addition to the improvement being limited in the Panorama paper, would make the level of improvement undetectable in the current study. Our patients were also treated predominantly with bevacizumab as compared to ranibizumab or aflibercept, which are more potent [15]. Our CTMC model did not allow for regression of DR. Additional investigations should be conducted to study the effect of anti-VEGF treatments in all DR patients with or without DME, but this is outside the scope of this study. This difference may not be generalizable to all populations and care systems. For example, the care system may delay the initial examination of new diabetics without DR, resulting in the appearance of a shorter sojourn time from no DR to any DR than expected.

### 4.2. Progression Time

Harris Nwayanyanu et al.'s study had a shorter progression time to PDR in the progressed patients (1.1 years) than that of our study and other studies [10–13]. This is likely due to the short follow-up time (1.7 years) in their study. Also, Harris Nwayanwu et al.'s study utilized the longitudinal Cox regression analysis for their data [10], and although both CTMC modeling and longitudinal Cox regression modeling can be used for estimating time-to-event with time-dependent risk factors, the Cox regression model is commonly used for 2-stage diseases (NPDR to PDR), while CTMC modeling can be used in the diseases with multiple stages, such as the 5 stages in DR. Thus, we believe that the CTMC model provides a more accurate prediction for each patient's condition. This could also potentially explain the shorter progression time in their study.

In a study by Tung et al., they followed 725 type 2 DM patients with a mean follow-up time of 2.56 ± 0.73 years. They studied the natural progression of DR using a 6-stage DR classification (no retinopathy, mild, moderate, severe NPDR, PDR, and blind) and found that the mean duration of DR in mild NPDR, moderate NPDR, and severe NPDR stages was 4.05 years, 4.18 years, and 2.52 years, respectively [13]. Our data showed a longer duration in each stage (4.63 years, 6.18 years, and 4.85 years for mild, moderate, and severe NPDR, resp.). There are several differences in terms of study population and methods between their study and ours. First, their study had a shorter follow-up period (3-year follow-up time with a mean of 2.56 years) [13] than ours (10-year follow-up time with a mean of 5.8 years). The shorter study period may lead to an underestimate of sojourn times. Second, our stage distribution was more evenly distributed when compared to that of Tung et al., which may lead to a more robust estimation. At baseline, Tung et al.'s study had 591 (81.5%) eyes with no retinopathy and only 64 (8.8%) with mild, 30 (4.1%) with moderate, and 15 (2.1%) with severe NPDR [13], while our study had 65 (28.3%) eyes with no retinopathy, 73 (31.7%) with mild, 60 (26.1%) with moderate, and 32 (13.9%) with severe NPDR. Third, their model did not adjust for any risk factors. Fourth, their model assumes that DR can only progress to the next stage between 2 consecutive visits while our model allows DR to progress to any advanced stage between 2 consecutive visits [13]. Finally, their study population was not as high risk compared to ours since their baseline HbA1c was 2.4% lower than ours. Their study population race/ethnicity was also all Asian (non-Hispanic) [13].

In the Srikanth study, 153 type 1 and type 2 DM patients with DR were enrolled in 2010 and were followed annually until 2013. Their 5-stage (mild, moderate, severe NPDR, PDR, and blind) homogeneous discrete-time Markov chain model showed that patients who entered mild NPDR were estimated to stay in the mild stage for 5 years. Those who entered the moderate NPDR stage were estimated to stay 1.78 years, and those who entered the severe NPDR stage were estimated to stay for 1.85 years [12]. Compared to our results, patients in Srikanth's study remained in the mild NPDR stage for 0.37 years longer. However, our results showed a 4.4-year longer duration in moderate NPDR and 3-year longer duration in severe NPDR. Similar to Tung et al.'s study, Srikanth's study had a shorter follow-up (3 years) and did not adjust for risk factors, which may explain the shorter progression time [12, 13].

In summary, after adjusting for risk factors, our progression time is longer than that of Tung et al.'s study conducted in Taiwan, Srikanth's study conducted in India, and Harris Nwayanwu et al.'s study conducted in the US [10, 12, 13]. All these previous studies had shorter follow-up times as well (1.7 years to 3 years), while the WESDR had a longer follow-up period. However, WESDR did not study progression time [11]. In terms of risk factors, both Tung et al. and Srikanth's studies did not investigate risk factors, while the WESDR and Harris Nwayanwu et al.'s studies focused on investigating the risk factors that affect DR progression or progression to PDR [10–13]. The consensus of the findings among these 2 studies and our study was that HbA1c is a significant risk factor for the progression of DR. In addition, both Harris Nwayanwu et al.'s study and our study showed that the age at initial visit had a protective effect on the progression of DR [10].

### 4.3. Limitations

Our study has several limitations. First, it is a retrospective study conducted at a teaching county hospital in which the majority of patients may have significant barriers to healthcare access. Second, patient noncompliance, lack of patient health literacy, and transportation limitations may affect our population. Race and ethnicity were self-identified, and the dataset did not clearly identify races of Hispanic individuals. This resulted in a group of patients driving a trend towards Hispanic ethnicity as a risk factor for progression. There is also a chance of observer bias, as this study is a retrospective study analyzing electronic medical records. Data were gathered from a single hospital site with a high Hispanic population which may not be fully representative of the demographics of the United States.

Despite these limitations, our study had a large sample size (*N* = 230) and followed patients for a mean of 5+ years, the second-longest follow-up time among the studies we reviewed and added to the risk factors of progression through NPDR stages.

## 5. Conclusions

We found that the increase in age and decrease in HbA1c levels were protective for the progression of DR. We also developed a model to predict the time and rate of DR progression for each stage based on each patient's risk factors. The proposed model could help clinicians customize an appropriate personalized follow-up schedule based on the patient's stage of disease and risk factors. Although race/ethnicity was not identified as a statistically significant risk factor, it is an important factor in the model. However, limitations in the dataset as stated above prevent us from concluding its importance in tailoring care to the patient. A prospective multicenter study should be conducted to further validate our model.

## Figures and Tables

**Table 1:** Transition rate matrix at time *t* of the 5-state model.

From	To				
	No retinopathy	Mild NPDR	Moderate NPDR	Severe NPDR	PDR
No retinopathy	−q11(*t*)	q12*t*	q13*t*	q14*t*	q15*t*
Mild NPDR	0	−q22*t*	q23*t*	q24*t*	q25*t*
Moderate NPDR	0	0	−q33*t*	q34*t*	q35*t*
Severe NPDR	0	0	0	−q44*t*	q45*t*
PDR	0	0	0	0	−q55*t*

NPDR = nonproliferative diabetic retinopathy; PDR = proliferative diabetic retinopathy.

**Table 2:** Summary of demographics and medical history.

Variable	Summary statistics (*N* = 230)
*Demographics*	
Age at initial eye exam (years, ±SD (range))	56.0 (±10.0) (20–84)
Sex (females (%))	143 (62.2%)
Race/ethnicity (%)	
White	20 (8.7%)
Black	50 (21.7%)
Hispanic	158 (68.7%)
Asian	2 (0.9%)

*Baseline systemic characteristics and medical history*	
Type of diabetes mellitus (type 2, %)	225 (97.8%)
Duration of diabetes mellitus (years, ±SD (range))	13.1 (±7.5) (0.8–38.7)
HbA1c level (%, ±SD (range))	9.7 (±2.4) (5.4–16.8)
Insulin-dependent (%)	62 (27.0%)
Smoking status (%)	
Previous smoker	40 (17.4%)
Current smoker	17 (7.4%)
Systolic blood pressure (mmHg, ±SD (range))	135.6 (±18.2) (91–205)
Diastolic blood pressure (mmHg, ±SD (range))	76.3 (±11.6) (51–111)
Hypertension (%)	184 (80.4%)
Hyperlipidemia (%)	172 (74.8%)
Presenting with systemic diseases (%)	43 (18.7%)
Myocardial infarction (%)	5 (2.2%)
Severe blockage requiring coronary artery bypass graft (%)	7 (3.0%)
Cerebrovascular accident (%)	8 (3.5%)
Chronic kidney disease (%)^1^	19 (8.6%)
Amputation (%)	12 (5.2%)

^1^Missing 10 data points.

**Table 3:** Summary of baseline ocular characteristics.

Variable	Summary statistics (*N* = 230)
*Nonproliferative diabetic retinopathy stage (%)*	
None	65 (28.3%)
Mild	73 (31.7%)
Moderate	60 (26.1%)
Severe	32 (13.9%)
Diabetic macular edema (%)	39 (17.0%)
Best-corrected visual acuity (logMAR, ±SD (range))	0.45 (±0.54) (0.0–2.6)
Glaucoma (%)	7 (3.0%)
Phakic eye (%)	215 (93.5%)
Cataract	144 (66.5%)
Age-related macular degeneration (%)	0 (0%)
Retinal detachment (%)	0 (0%)
Hypertensive retinopathy (%)	2 (0.9%)
Retinal artery occlusion (%)	0 (0%)

SD = standard deviation.

**Table 4:** Summary statistics for variables collected during the follow-up period by year.

Visit	DR progressed *n* (%)	HbA1c mean (±SD)	SBP mean (±SD)	DBP mean (±SD)	DME *n* (%)	Anti-VEGF treatment for DME *n* (% DME eyes)	Number of anti-VEGF treatments for DME eyes mean (±SD)
Baseline (*N* = 230)	—	9.71 (±2.35)	135.6 (±18.2)	76.3 (±11.5)	39 (17%)	3 (8%)	—
Year 1 (*N* = 157)	23 (15%)	9.17 (±1.90)	135.4 (±13.6)	75.2 (±8.2)	49 (31%)	26 (53%)	2.9 (±1.5)
Year 2 (*N* = 183)	46 (25%)	8.88 (±1.98)	136.6 (±13.1)	74.6 (±9.2)	72 (39%)	34 (47%)	3.5 (±1.8)
Year 3 (*N* = 175)	43 (25%)	8.82 (±1.86)	138.6 (±13.0)	73.9 (±9.7)	82 (47%)	39 (48%)	2.6 (±1.7)
Year 4 (*N* = 168)	27 (16%)	8.89 (±1.96)	138.0 (±13.0)	73.1 (±9.0)	75 (45%)	30 (40%)	3.2 (±2.0)
Year 5 (*N* = 147)	32 (22%)	8.97 (±2.08)	137.2 (±12.2)	72.9 (±10.5)	66 (45%)	22 (33%)	3.0 (±2.3)
Year 6 (*N* = 123)	19 (15%)	8.76 (±1.79)	137.6 (±12.6)	72.1 (±11.1)	53 (43%)	18 (34%)	3.4 (±2.3)
Year 7 (*N* = 91)	14 (15%)	8.71 (±1.82)	137.9 (±12.7)	72.8 (±11.2)	51 (56%)	12 (24%)	3.4 (±1.9)
Year 8 (*N* = 78)	7 (9%)	8.71 (±1.91)	138.4 (±11.2)	72.1 (±11.9)	41 (53%)	10 (24%)	2.5 (±1.4)
Year 9 (*N* = 38)	4 (11%)	8.59 (±1.59)	134.9 (±12.0)	70.6 (±17.3)	22 (58%)	7 (32%)	2.1 (±1.2)
Year 10 (*N* = 10)	0 (0%)	8.16 (±0.93)	135.7 (±5.1)	73.4 (±7.1)	7 (70%)	3 (43%)	2.0 (±1.0)

DR = diabetic retinopathy; SBP = systolic blood pressure; DBP = diastolic blood pressure; DME = diabetic macular edema; anti-VEGF = antivascular endothelial growth factor; SD = standard deviation.

**Table 5:** Effect of time-independent risk factors in the final model.

Time-independent risk factor	Hazard ratio	95% confidence interval	*P*
Age (per year)	0.99	(0.973–0.999)	0.047
Duration of DM (per year)	1.02	(1.000–1.039)	0.018
Hispanic (versus non-Hispanic)	1.31	(0.969–1.767)	0.068

DM = diabetes mellitus.

**Table 6:** Average sojourn time (±SD) and the effect on HbA1c for each stage.

Stage	Average sojourn time (years)	When HbA1c increased by 1 per year	
		% sojourn time reduction	Years of sojourn time reduction
No retinopathy	3.03 (±0.97)	15	0.46
Mild NPDR	4.63 (±1.21)	10	0.47
Moderate NPDR	6.18 (±1.45)	7	0.46
Severe NPDR	4.85 (±1.25)	10	0.47

NPDR = nonproliferative diabetic retinopathy; SD = standard deviation.

**Table 7:** Estimated average transition rate matrix over time (±SD) for a non-Hispanic individual, assuming that other risk factors take their mean values.

From	To			
	Mild NPDR	Moderate NPDR	Severe NPDR	PDR
No retinopathy	0.209 (±0.044)	0.068 (±0.026)	0.003 (±0.008)	<0.001^*∗*^ (±0.002)
Mild NPDR	—	0.142 (±0.026)	0.013 (±0.010)	0.027 (±0.010)
Moderate NPDR	—	—	0.097 (±0.020)	0.034 (±0.012)
Severe NPDR	—	—	—	0.172 (±0.036)

^*∗*^Estimated 0.0004; NPDR = nonproliferative diabetic retinopathy; PDR = proliferative diabetic retinopathy; SD = standard deviation.

**Table 8:** Probability of transitioning to proliferative diabetic retinopathy within 1, 4, and 7 years.

From	Probability to PDR (%)	95% confidence interval
*Within 1 year*		
No retinopathy	0.7	(0.46–31.05)
Mild NPDR	3.5	(2.14–6.16)
Moderate NPDR	5.0	(3.29–8.26)
Severe NPDR	19.3	(14.26–26.17)

*Within 4 years*		
No retinopathy	8.0	(6.26–63.78)
Mild NPDR	16.0	(12.06–23.89)
Moderate NPDR	24.1	(18.62–32.58)
Severe NPDR	56.4	(44.88–68.68)

*Within 7 years*		
No retinopathy	20.6	(16.99–73.40)
Mild NPDR	30.8	(24.95–41.64)
Moderate NPDR	43.6	(35.02–54.08)
Severe NPDR	76.4	(64.24–86.74)

NPDR = nonproliferative diabetic retinopathy; PDR = proliferative diabetic retinopathy.

## Data Availability

The dataset for this study can be accessed at https://figshare.com/s/849a4b0a2b193d44cbc6 (private link for review).

## Supplementary Materials

Supplementary Materials S1. Nonhomogeneous continuous-time Markov chain model. S2. Estimation of sojourn time. S3. Estimation of transition probability matrix.
