# Supplementary material for: Determining Risk Factors That Affect Progression in Patients with Nonproliferative Diabetic Retinopathy
Source: J Ophthalmol. 2021 Nov 30;2021:6064525. doi: 10.1155/2021/6064525 (PMC8651358; doi:10.1155/2021/6064525)
Supplement: Supplementary Materials — S1. Nonhomogeneous continuous-time Markov chain model. S2. Estimation of sojourn time. S3. Estimation of transition probability matrix. [file 6064525.f1.docx]

**S1.** *Nonhomogeneous Continuous-Time Markov chain Model*

A 5-state nonhomogeneous Continuous-Time Markov chain (CTMC) was used to analyze the progression of diabetic retinopathy (DR). The CTMC model was used to estimate the transition rate matrix $Q(t)$ (Table 1) and the effects of risk factors. This model assumes that

1. The progression of DR is irreversible; hence transitions where the disease stage moves from a higher to a lower stage were not allowed.
2. DR is allowed to progress without going through the intermediate stages, such as transitioning from mild to severe stages without going through the moderate stage.
3. The transition (progression) rate from stage $i$ to $j$ at time $t$ is denoted as $q_{ij}\left( t \right)$.
4. The effects of risk factors, $\beta,$ are independent of the disease stages, meaning that the risk factors affect the whole transitioning process by a common factor.
5. The time-dependent risk factors are constant between observation times, e.g. between year 1 and year 2, HbA1c value is same as the value obtained at year 1 visit.
6. The sojourn time at stage$i$, $T_{i}$, is the amount of time spent in the stage$i$ before progression to another stage. $T_{i}$ is exponentially distributed under the Markov property.

The elements in Table 1 can be expressed as the following^1-3^:

$$q_{ij}\left( t \right)=r_{ij} exp \left( \beta^{T}x\left( t \right) \right) , for i\neq j, and {-q}_{ii}\left( t \right)= -\sum_{j: i\neq j} q_{ij}\left( t \right) for i=j$$

where $x(t)$ is a vector of risk factors at time $t$, $\beta$ is a vector of the corresponding effects, and $r_{ij}$ are the baseline progression rates, i.e. progression rates when all risk factors take the value of zero, which is analogous to the intercept term in regression models. The "msm" R package (version 1.6.8) was utilized to obtain the maximum likelihood estimation of transition rates, $q_{ij}\left( t \right).$

**References**

1. Jackson CH. Multi-state models for panel data: the msm package for R. J Stat Software 2011;38(8):1-29.

2. Kalbfleisch JD, Lawless JF. The analysis of panel data under a Markov assumption. J Am Stat Assoc 1985;80(392):863-71.

3. Kay R. A Markov model for analysing cancer markers and disease states in survival studies. Biometrics 1986;42(4):855-65.

**S2.** *Estimation of Sojourn Time*

We estimated the sojourn time by first deriving the cumulative density function (CDF) of $T_{i}$ as

$$P\left( T_{i}>t \right)=exp \left( -\int_{0}^{t} q_{ii}\left( \tau\right)d\tau\right) =exp\left( -\int_{0}^{t} r_{ii} exp \left( \beta^{T}x\left( \tau\right) \right) d\tau\right)$$

Therefore, the expected sojourn time of stage-$i$ is

$$E\left( T_{i} | x\left( t \right) \right)=\int_{0}^{\infty} P\left( T_{i}>t \right)dt= \int_{0}^{\infty} exp \left( -\int_{0}^{t} r_{ii} exp \left( \beta^{T}x\left( \tau\right) \right) d\tau\right) dt.$$

Since time-dependent risk factors are assumed constant between observational times, the mean sojourn time of stage-$i$ was derived using a piecewise integration approach

$$E\left( T_{i} | x\left( t \right) \right) \approx\sum_{m=0}^{n-1} \int_{t_{m}}^{t_{m+1}} exp \left( -\int_{t_{m}}^{t} r_{ii}exp \left( {\frac{1}{2}\beta}^{T}\left( x_{m}+x_{m+1} \right) \right) d\tau\right) dt$$

Where $x_{0}, \ldots, x_{n}$ represents the vector of risk factors observed at time $t_{0},\ldots, t_{n}$. When $t_{n}$is large (>20), values of $t_{n}$ the integrals under the summation are close to zero, and, hence, truncating the tail portion of the infinite sum should keep our accuracy of expected $T_{i}.$

**S3.** *Estimation of Transition Probability Matrix*

In homogeneous CTMC, the transition probability matrix from time $t_{1}$ to $t_{n}$ is $P\left( t_{1},t_{n} \right)=e^{Q*\left( t_{n}-t_{1} \right)}$, where $Q$ is a time-independent progression rate matrix. By the piecewise constant assumption, the progression probability matrix in our nonhomogeneous CTMC model, $P\left( t_{1},t_{n} \right),$can be expressed as the product of several homogeneous segments as the following:

$$P\left( t_{1},t_{n} \right)=P\left( t_{1},t_{2} \right)\cdot\ldots\cdot P\left( t_{n-1},t_{n} \right)=exp \left( Q\left( t_{1} \right)\times\left( t_{2}-t_{1} \right) \right)\cdot\ldots\cdot exp \left( Q\left( t_{n-1} \right)\times\left( t_{n}-t_{n-1} \right) \right)$$

For example, the probability of progression from mild DR (stage 2) to PDR (stage 5) from baseline to year 4 of the study can be estimated as the $p_{25}$ component of the matrix $P\left( t_{1}=0, t_{n}=4 \right)$.
